# Setting up a rapid diagnostic clinic for patients with vague symptoms of cancer: a mixed method process evaluation study

**DOI:** 10.1186/s12913-021-06360-0

**Published:** 2021-04-17

**Authors:** Christos Vasilakis, Paul Forte

**Affiliations:** Centre for Healthcare Innovation and Improvement (CHI2), School of Management, University of Bath, Bath, UK

**Keywords:** Cancer diagnosis, Early detection of cancer, General practice, Non-specific symptoms, Rapid Diagnostic Clinic

## Abstract

**Background:**

The study sought to evaluate the impact of a Rapid Diagnostic Clinic (RDC) service designed to improve general practitioner (GP) referral processes for patients who do not meet existing referral criteria yet present with vague - but potentially concerning - symptoms of cancer. We sought to investigate how well the RDC has performed in the views of local GPs and patients, and through analysis of its activity and performance in the first two years of operation.

**Methods:**

The study setting was a single, hospital-based RDC clinic in a University Health Board in South Wales. We used a mixed-method process evaluation study, including routinely collected activity and diagnosis data. All GPs were invited to participate in an online survey (34/165 responded), and a smaller group (*n* = 8) were interviewed individually. Two focus groups with patients and their carers (*n* = 7) provided in-depth personal accounts of their experiences.

**Results:**

The focus groups revealed high rates of patient satisfaction with the RDC. GPs were also overwhelmingly positive about the value of the RDC to their practice. There were 574 clinic attendances between July 2017 and March 2019; the mean age of attendees was 68, 57% were female, and approximately 30% had three or more vague symptoms. Of those attending, we estimated between 42 to 71 (7.3 and 12.3%) received preliminary cancer diagnoses. Median time from GP referral to RDC appointment was 12 days; from GP referral to cancer diagnosis was 34 days. Overall, 73% of RDC patients received either a new diagnosis (suspected cancer 23.2%, non-cancer 35.9%) or an onward referral to secondary care for further investigation with no new diagnosis (13.9%), and 27% were referred to primary care with no new diagnosis.

**Conclusions:**

The RDC appears to enable a good patient experience in cancer diagnosis. Patients are seen in timely fashion, and the service is highly regarded by them, their carers, and referring GPs. Although too early to draw conclusions about long-term patient outcomes, there are strong indications to suggest that this model of service provision can set higher standards for a strongly patient-centred service.

**Supplementary Information:**

The online version contains supplementary material available at 10.1186/s12913-021-06360-0.

## Background

The process of care for cancer patients, from diagnosis to treatment, is often fragmented requiring a number of interactions with different care professionals in primary, secondary and tertiary care [1]. Some patients are only identified at a late stage in the disease progression through an emergency hospital admission, while others may first present with first (or later) stage symptoms in primary care. Partly in response to this variation, urgent suspected cancer pathways for assessment of patients with symptoms that raise suspicion of cancer in a specific site have been in place for a number of years, for example, for suspected colorectal, lung, ovarian and prostate cancer [2]. However, less than 40% of cancers in Wales are diagnosed in this way, while in some places this figure is even lower at around 35% [3]. Additionally, many of the patients who go on to receive a diagnosis of cancer initially present with non-specific or vague symptoms such as fatigue, loss of appetite or unexplained weight loss. Although these are symptoms which warrant investigation [4], they do not readily align with referral criteria for many cancers when presented in isolation and in the absence of a specific lump, bleeding or more physically alarming symptom. In the absence of specific pathways for this group of patients [5], unnecessary and unacceptable delays in diagnosis can occur [6].

As part of the Welsh Government National Cancer Delivery Plan, two ‘Rapid Diagnostic Clinic’ (RDC) pilot projects were established at Cwm Taf University Health Board (UHB), and at Abertawe Bro Morgannwg UHB (ABM; see [7] for more details). Patients with concerning vague or non-specific symptoms often do not fit existing Urgent Suspected Cancer referral criteria and, where the underlying condition is less obvious, the general practitioner (GP) may have to coordinate a series of diagnostic investigations, choose a specialty to refer to, or decide on a period of ‘watchful waiting’ to see if more specific symptoms develop before making a referral decision. A combination of these factors can result in delayed cancer diagnoses, unnecessary investigations and consultations being performed, unnecessary or prolonged distress for the patient [8] and, ultimately, potentially poorer long-term clinical outcomes.

A single cancer pathway from primary care to Multidisciplinary Team (MDT) with diagnostics at an early stage is seen as an appropriate model to use, and the pilot RDCs have been established as a way of implementing this concept. It provides a rapid access clinic where a range of diagnostic tests are conducted on a single visit (whenever possible), and where several specialists work together to speed up diagnosis for the patient. The RDC model of care was first developed in Denmark where research indicated that 30% of first cancer presentations in general practice would be categorised as ‘vague symptoms’ [9]. This led to the development of the RDC model where GPs could refer these types of patients for diagnostic investigations to ascertain if they were symptomatic of cancer [10, 11]. Recent evidence suggests that mortality among cancer patients examined in such diagnostic centre is comparable to cancer patients diagnosed through other routes [12].

The RDC pathway targets patients presenting to their GP with vague, ‘non-specific’ symptoms (especially fatigue and weight loss, Table 1) but which nevertheless cause the GP concern and leads them to suspect that there might possibly be an underlying cancer diagnosis, even in the absence of obvious cancer ‘red flags’. The RDC provides rapid diagnoses for patients referred by GPs with ‘vague symptoms’ which they suspect may be indicative of cancer. In streamlining and accelerating the cancer diagnosis process in this way it is also the intention to capture more cancer diagnoses at an early clinical stage.

**Table 1:** Working definition of ‘vague symptoms’ in Cwm Taf

Abdominal pain	
Unexplained weight loss	
Fatigue	
Mild anaemia	
Shortness of breath	
Nausea	
Lack of appetite	
Unexpected lab result	
GP ‘gut feeling’	

The RDC, which was launched in stages in each of the four UHB localities between July 2017 and February 2018, operates from the Diagnostic Hub within the Royal Glamorgan Hospital. The service is open to referrals from all Cwm Taf GP practices for patients over the age of 18 who do not meet the criteria for an Urgent Suspected Cancer pathway. The referral relies on GP ‘gut feeling’; if the patient is experiencing non-specific symptoms and the GP suspects an underlying cancer diagnosis, they can be referred to the service.

As part of the RDC referral protocol, GPs request a suite of 11 tests (e.g. bloods, urine) and a chest X-ray at the point of referral so results are available when the patient attends the clinic. All referrals are initially vetted by a Consultant Physician following their receipt in secondary care to ensure they are suitable for the pathway and do not require redirection to a site-specific pathway. Once the test results are available on the Welsh Clinical Portal [13], the patient is booked into the next available RDC clinic slot by the Clinic Coordinator, with the intention that the patient is seen within 14 days.

Clinics are held on two separate half-days each week. There are two parallel sessions which enable a total of 15 appointment slots each week. On arrival at the clinic, the patient is seen by either a Consultant Physician, GP, or Advanced Nurse Specialist where they undergo a physical examination and medical history prior to having a CT scan (thorax/abdomen and pelvis). The CT scan is reported live by a Radiologist and the results and a management/ treatment plan discussed with the clinician. The results are explained, in turn, to the patient and any relevant onwards referrals made. At this point the patient is discharged from the RDC service. The entire clinic appointment lasts approximately 2 h and there are three main outcomes: suspected cancer (with a referral to the cancer MDT, or for further investigation); a non-cancer diagnosis (with a referral to an appropriate secondary care clinic, or back to GP); or nothing specific is diagnosed (usually referred back to GP, although a secondary care referral for further tests is also possible).

The multi-disciplinary RDC team is led by a consultant respiratory physician and at the time of the study included the following team members:

- Consultant physicians (2)
- Radiologist (3)
- Advanced Nurse Practitioner (1)
- GPs (3)
- Clinic Coordinator (2)
- Healthcare Support Worker (1)
- Management support (provided by the Radiology Directorate and Planning Department)

Our study was driven by two main questions:

1. How well has the RDC performed in the views of local GPs and patients?
2. What has the activity and performance of the RDC been in the first 2 years of its operation?

## Methods

All analyses were carried out on data and boundaries associated with Cwm Taf UHB prior to 1 April 2019 (when it became Cwm Taf Morgannwg). Unless otherwise stated, analyses are based on data up to 31st March 2019.

### Patient engagement focus groups

Two focus groups were held with patients and their supporters (usually close family members) where all described their experiences of the RDC in depth. This included the background to their condition, and their care and support journey from initial treatment of symptoms in primary care through to RDC referral. The patients were approached by RDC managers to take part if they so wished. Each focus group was facilitated by the two authors with a Cwm Taf staff member also in attendance and lasted about 90 min.

### GP engagement

As GPs are the sole point of referral to RDC, their use and experience of it as an effective clinical service is crucial to its success and it was essential to obtain their views on this. We approached this in two ways:

- Through an online questionnaire sent via Practice Managers giving all Cwm Taf GPs an opportunity to respond.
- A more detailed evaluation via a series of one-to-one, semi-structured interviews with selected GPs (conducted by the authors).

The online survey comprised multiple-choice questions taking about 10 min to complete and with options to comment further for each - and many did so. Stratification of the interview sample reflected a range of practice sizes, location, and frequency of RDC referrals.

Each face-to-face interview was conducted by both or one of the two male co-authors with experience in conducting qualitative interviews and lasted between 30 and 45 min. Participants were approached via telephone and/or email and the interviews took place in their practices (apart from one that took place in a public space). Detailed notes were taken during the interviews, which were not audio or video recorded, and no one else was present besides the participants and the researchers. This provided an informal atmosphere for discussion without unduly compromising the quality of the information obtained [14]. Interview notes were sent to the interviewees via email to solicit their comments and feedback. Prior to the interviews, neither of the authors was known to the participants and therefore they had no pre-existing relationship with them. Participants were informed about the study both verbally and in writing using a participant information sheet. No characteristics about either of the interviewers were reported. Both the questionnaire and interview guide, which was developed for this study and was pilot tested in the first interview, can be found in the supplementary file. A hybrid approach to thematic analysis was used, whereby themes were identified directly from the data (inductively), and by considering the aims of evaluation (deductively) [15]. Data analysis was carried out by both researchers.

### Routinely collected data

We used several anonymised datasets provided by the Cwm Taf UHB information team. Specifically:

1. An electronic dataset, maintained within the RDC, of all patient referrals received from inception until 31st March 2019. This included all patients who attended the clinic as well as those whose referral had been received although they may not have attended before 31st March). Also included were referrals clinically reviewed in the RDC but deemed not necessary to attend. Data recorded in this dataset includes basic patient demographic information (age, gender), referral and appointment dates, specialist seen, patient diagnoses and outcomes (in terms of further referrals).
2. Routinely collected hospital activity data for those patients known to have had an RDC appointment between 1 July 2017 and 31 March 2019 (i.e. from the inception of the RDC to the end date of the evaluation period). These included all patient referrals to the clinic who attended, as well as those who were invited, but did not attend (e.g., due to personal reasons or hospital cancellation) and for whom we could not find a subsequently re-arranged and attended RDC appointment. This dataset did not include referrals that were reviewed but not invited to attend the RDC clinic.
3. Routinely collected hospital data for cancer patients from the Cancer Network Information System Cymru (CaNISC) which is a national cancer patient tracking system for Wales.

Significant effort was required in data preparation prior to analysis which included: cross-referencing records across the different datasets; accounting for duplicate records (some represented multiple RDC attendances by the same patient); accounting for mis-typed hospital numbers (crucial to linking data items across different activity data sets); referral and treatment dates not correctly connected with appointment dates. We also supported local codification of free-text data fields in the local RDC dataset to enable quantitative analysis.

The linked evaluation dataset was used to conduct descriptive statistical analysis (mean, standard deviation, proportion, median, and interquartile range) and to calculate the following metrics: preliminary cancer diagnosis rate (the number of patient with a cancer diagnosis over the number of patients attending the RDC; sometimes referred to as the cancer conversion rate); the median times and interquartile range (IQR) from GP referral RDC appointment and from GP referral to cancer diagnosis.

### Costs

Given the documented challenges with conducting cost effectiveness analysis in this setting [16] and as the RDC was already in operation when our study commenced, we adopted a pragmatic design focusing on direct RDC fixed staff costs averaged over first attendances, and marginal treatment costs related to the number of patient first attendances. As we were limited to routinely available data, these figures are illustrative rather than definitive as they do not, for example, include management or estate overhead costs.

## Results

### Patient engagement

We organised and facilitated two patient focus groups attended by seven participants in total; patients (*n* = 4) accompanied by partners or relatives (*n* = 3). Both groups were ‘very satisfied’ with their experiences of the RDC process and the sensitive manner with which potentially very difficult results and information were presented. There was sufficient time for clear explanations about the implications of the diagnosis, and direct involvement of patient supporters/ carers throughout. The speed of the referral process was particularly highly valued as it lessened ‘worry time’ for patients and carers. All said they had no hesitation in recommending the RDC to any friends or family with similar symptomatic conditions to proactively seek a referral to the RDC service (and one had actively done so). Specific suggestions for further improvements were offered including:

- Ensuring good communication channels between GPs, the RDC and other hospital departments
- More information about the RDC for patients at GP practices
- Additional RDC clinic sessions to enable more patients to benefit from the service

#### Case study vignette – patient A

- An active adult, this person had noticed getting tired and short of breath in September 2018 with fatigue and weight loss (more than 2 kg in 1 month) as principle symptoms.
- After a further recurrence of fatigue, they attended their GP in February 2019. Blood tests and an X-ray were inconclusive but, after returning to their GP in March 2019 with similar symptoms, they were referred to the RDC which they attended in April 2019. The overall experience was positive, reassuring and efficient from point of arrival. After initial body measurements they were examined by a doctor and had a CT scan where the radiographers explained everything clearly.
- After a short break, they returned to the clinic to be given a ‘no cancer’ diagnosis, but ‘background emphysema’ and gallstones were reported and a referral for ultrasound was made at that time (at the time of interview, this follow-up was still awaited). Despite still having some symptoms, there was relief at having a likely diagnosis and having results on the same day was very important for them.

#### Case study vignette – patient B

- This adult had recurring urinary tract infections over a 3 month period in early 2018 which did not respond to antibiotics. Their GP suggested referral to the RDC, which was attended 10 days later in early summer 2018 following all pre-clinic preliminary tests.
- They had an ‘excellent explanation’ by the clinician on the initial consultation at the clinic about what to expect and, following the CT scan, received a diagnosis of a likely non-Hodgkin’s lymphoma cancer at the end of the afternoon. They felt that there was plenty of time given to explain what that meant for themselves and their spouse, with good reassurance as to the potential for treatment and that there was no cause for panic.
- A follow-up PET scan also revealed a small malignant growth in the lung; six courses of chemo later and they feel ‘mainly OK’ now.
- Both the patient and their spouse attribute a large part of this success from the reassurance given, and from knowing the nature and extent of the condition in a very short time rather than weeks of waiting.
- “I don’t think I would have been diagnosed and treated so quickly if that clinic didn’t exist”. “It saved my life”.

### GP engagement

We undertook two forms of GP engagement: direct interviews with eight GPs on their experience of referring patients to the RDC, and an online survey sent to all GP practices which was completed by 34 /165 GPs (21%), with 11 out of 34 respondents (32%) also providing valuable supplementary commentary. Practices from all UHB localities responded to the survey.

Online survey results indicated that GPs were overwhelmingly positive about the RDC with 88% of respondents either satisfied or very satisfied, and often coupled with strongly supportive additional comments (‘I think this clinic has made a big difference to my practice’).

Aspects of the RDC service (such as clarity of referral criteria; ease of the referral process; speed with which patients were seen; quality of information fed back to GPs) were also highly rated (typically 80–90% of respondents were ‘satisfied’ or ‘very satisfied’). However, certain aspects were less positively viewed: 18% of respondents reporting that there were ‘very often’ problems or significant delays in ordering pre-RDC attendance tests (difficulties in the specimen collection service from practices were mentioned by several respondents, for example, a practice did not have access to a daily transportation service of samples to a laboratory).

An interesting finding concerned levels of ‘RDC awareness’ within primary care. Among GP principals this was ‘high’ or ‘very high’ (94%), dropping to 59% for trainees, and only 10% for locums.

Results from the GP interviews also demonstrated overwhelming support for the RDC and the value it provided for clinicians and patients. Benefits frequently mentioned included:

- Speed of referral and diagnosis
- Ease of access for diagnostic testing (especially CT scans)
- Straightforward referral process
- Reduction of stress for GPs in being able to refer patients for vague symptoms.

The last point was particularly notable. GPs were not only concerned about delays in diagnosis and back-and-forth referral and testing for their patients, but also at the ‘loss of professional face’ they sometimes previously experienced when they had referred a patient with vague symptoms only to have them rejected as an inappropriate referral to that particular specialty. With the RDC that potential stigma is removed.

Rapid access to diagnostic testing was seen as particularly helpful. However, issues were mentioned over having pre-appointment tests undertaken in time (by the GP), and the report-back process also came in for some negative comment. However, we understand anecdotally that this can be due to the way in which reports are managed within practices themselves rather than the speed of reporting back to them from the RDC itself.

Specific recommendations about the RDC made by GPs included:

1. Continuous promotion of awareness of the RDC, especially to support locums and trainees.
2. Introduce more feedback to primary care about RDC performance such as waiting times and outcomes.
3. Introduce pre-printed haematology and microbiology blood forms specifically for RDC referral tests and develop an electronic referral form accessible from GP systems with reminders about tests that could be auto-populated with patient details.
4. Consider extending the clinic for the rapid diagnosis from suspected cancer to other diseases and conditions (e.g. pulmonary fibrosis or abdominal pain).
5. Review the requirement for pre-RDC tests for patients.

### Process and outcome evaluation of RDC activity

Between July 2017 and 31st March 2019, 681 referrals resulted in 574 (84%) attendances (the introduction of the RDC was staggered across four localities; starting with Cynon in July 2017 and finishing with Taf Ely in February 2018), Table 2. There were 4 hospital cancellations, 6 patient cancellations, 3 patients who did not attend their appointment, and 94 referrals that were either not required to attend (as deemed by the senior RDC clinician) or had been referred, but were yet to receive, an RDC appointment.

**Table 2:** Patients referred to the Rapid Diagnostic Clinic (RDC) between 1st July 2017 and 31st March 2019

	Patients referred to RDC (***N*** = 681)	
All referrals, n (%)		
Attended	574	(84.3)
Hospital cancellation	4	(0.6)
Patient cancellation	6	(0.9)
Patient did not attend (DNA)	3	(0.4)
Appointment not offered	94	(13.8)
Appointment not offered, n (%) [*N* = 94]		
Awaiting scheduling of appointment	26	(27.7)
Admitted to hospital	15	(16.0)
Referred to other cancer pathway	14	(14.9)
Referred to other non-cancer pathway	14	(14.9)
Referred back to GP	10	(10.6)
Patient declined/could not be contacted	7	(7.4)
Other/unknown	5	(5.3)
Deceased	3	(3.2)

Of those attending an RDC appointment, the average age was 68 (68.3 for women; 67.6 for men); 58% were female; and approximately 30% had three or more vague symptoms, Table 3. The mean number of symptoms recorded by the referring GP dropped from 2.83 at the start of the operation of the RDC, to 1.53 symptoms in Quarter 1, 2019. ‘Weight loss’ was by far the most common symptom and there was a strong association between that and ‘lack of appetite’ with 100 patients presenting with both symptoms.

**Table 3:** Patients attending the Rapid Diagnostic Clinic (RDC): demographics and referral symptoms

Demographics	Patients attending RDC (***N*** = 574)	
Age at appointment		
Mean (SD)	68.0	(14.2)
Median (IQR)	71	(60, 79)
Sex, n (%)		
Female	331	(57.7)
Male	243	(42.3)
**Referral symptoms**		
Number of symptoms on referral letter, n (%)		
0	1	(0.2)
1	203	(35.4)
2	196	(34.1)
3	111	(19.3)
4+	63	(11.0)
Symptom type, n (%) [*N* = 1198]		
Weight loss	438	(36.6)
Fatigue	153	(12.8)
Lack of appetite	113	(9.4)
Unexpected lab result	80	(6.7)
Abdominal pain	74	(6.2)
Nausea	60	(5.0)
Anaemia	55	(4.6)
Shortness of breath	30	(2.5)
Other	195	(16.3)

*RDC* Rapid Diagnostic Clinic, *SD* Standard Deviation, *IQR* Interquartile Range

The local RDC database contains outcomes following the patient visit, Table 4. Of those, 59 patients (10.3%) were referred onwards to a specialist cancer pathway (directly to the Multi-Disciplinary Team meeting stage), and an additional 74 patients (12.9%) had a suspected cancer with further investigations deemed necessary. Almost a quarter of patients (128, 22.3%) were given a new, non-cancer diagnosis and referred to specialists in secondary care for further review and treatment. A further 78 patients (13.6%) received a new, non-cancer diagnosis but were referred back to their GP for evaluation and treatment. The remainder received no new diagnosis and were either referred to secondary care (80, 13.9%), or back to their GP (155, 27%).

**Table 4:** Recorded outcomes of patients attending the Rapid Diagnostic Clinic (RDC)

Outcomes	n	%
A1 - Suspected Cancer (to MDT)	59	10.3
A2 - Suspected Cancer (further investigations)	74	12.9
B1 - Non-cancer diagnosis (to secondary care)	128	22.3
B2 - Non-cancer diagnosis (back to GP)	78	13.6
C1 - No new diagnosis (to secondary care)	80	13.9
C2 - No new diagnosis (back to GP)	155	27.0
*Total*	*574*	*100.0*

*MDT* Multi-Disciplinary Team, *GP* General Practitioner

For the purposes of this evaluation we assumed that all ‘A1’ outcomes indicated a cancer diagnosis (*n* = 59) and a brief review of additional information highlighted at least 12/ 74 of the ‘A2’ outcomes as also potentially indicative of cancer, giving an estimated total of 71 patients seen at the RDC with a strong suspicion of cancer (with all cancer diagnoses subject to confirmation by biopsy).

We also analysed an extract of routinely collected hospital data for cancer patients which is compiled at an all-Wales level (CaNISC; see routinely collected datasets in the Methods section and glossary). As there is a time lag before patient records appear in this database, we did not expect to obtain all confirmed cancer diagnoses following an RDC appointment, but we were able to identify 42 patients with a confirmed cancer diagnosis following their RDC clinic attendance.

Based on the above data, two different preliminary cancer diagnosis rates were calculated: 7.3% (42/574) and 12.3% (71/574), which represent the likely lower and upper bounds of the RDC ‘cancer diagnosis’ rate.

Using the CaNISC data for the 42 confirmed cases, we identified the tumour site for these patients as well as the treatment they received (Table 5). Gastro-oesophageal cancers were the largest group (21%), followed by urological and lung cancers (both 17%), and colorectal and haematological cancers (12% each).

**Table 5:** Primary tumour site and types of treatment for Rapid Diagnostic Clinic (RDC) patients diagnosed with cancer (*N* = 42)

Primary tumour site	N	%
Gastro-Oesophageal	9	21.4
Urological	7	16.7
Lung	7	16.7
Unknown primary	6	14.3
Colorectal	5	11.9
Haematological	5	11.9
Breast	2	4.8
Gynaecological	1	4.8
**Total**	**42**	**100.0**
**Treatment**	**N**	**%**
Palliative care	13	31.0
Active monitoring	6	14.3
Surgery	6	14.3
Radiotherapy	5	11.9
Chemotherapy	4	9.5
Hormone therapy	3	7.1
Other	3	7.1
Unknown	2	4.8
**Total**	**42**	**100.0**

A key RDC objective is to identify non-cancer diagnoses (B1 and B2) as well as cancers in patients referred with vague symptoms. Overall, 73% of patients seen received either a diagnosis (cancer or non-cancer) and/or an onward referral to secondary care, (i.e. all outcomes except C2).

**Fig. 1:**
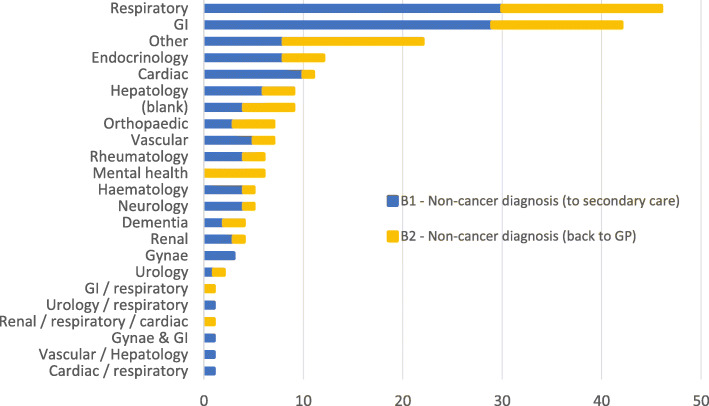
RDC patients with non-cancer onward referrals by ‘diagnosis group’ (*n* = 206). Blank: no diagnosis recorded in the dataset

A record of subsequent non-cancer diagnoses is not maintained, but we undertook a classification of the type of condition based on a text field analysis of the diagnosis and specialty of onward referral where known (Fig. 1). Respiratory (*n* = 46 onward referrals) and GI-related referrals (*n* = 42) were the most common (whether to secondary care or GP). Several classified as ‘mental health’ (*n* = 6) relate to drug and alcohol addiction; these were all referred back to the GP. Conversely, cardiac referrals (*n* = 11) are nearly all to secondary care. Endocrinology (*n* = 12) includes several patients where their diabetes appeared to be out of control. Several referrals appear to be for (potentially) two different types of diagnosis.

Finally, median time to RDC appointment from GP referral was 12 days (IQR 7, 19) and median time from GP referral to cancer diagnosis was 34 days (IQR 13, 50).

### Costs

There are two main cost dimensions: fixed (e.g. staff) and marginal (e.g. scans, tests). Fixed costs do not vary in the short to medium term and their unit cost thus decreases as increasing numbers of patients are seen within the available capacity (15 patients per week over two sessions on a Tuesday afternoon and a Thursday morning). Additional capacity would require a step-change increase in fixed costs for more clinic sessions and staff time. Marginal unit costs, however, increase directly with the number of patients seen with every extra patient generating additional expenditure.

Patients attend the clinic for 2–3 h altogether, at least 1–2 h of which is time waiting between the initial consultation and scan for the results and follow-up final consultation and diagnosis. From relevant 2018/19 cost outturn data we derived a staff cost per first attendance of each patient (£321). This includes all direct clinical and non-clinical staff time ascribed to the RDC (which includes consultant radiology and RDC clinicians). We were also provided with marginal cost data associated with undertaking blood tests and radiology examinations in the hospital (£185 per patient first attendance). All patients undergo the same suite of investigations irrespective of clinical presentation. However, we were not able to obtain any RDC clinic overhead costs, so our estimated total cost per first patient attendance – £506 – is an under-estimate.

## Discussions

### Summary of findings

In this paper, we report on a process evaluation study that explored the initial impact of a new RDC referral pathway on patient and staff experience and on process outcomes using a mixed-method, pragmatic study design. More and earlier stage cancer detection and improved long-term survival rates are key objectives of the RDC model. Although these objectives could not be evaluated per se, our study found that the RDC pathway enables a good patient experience in cancer diagnosis. Patients are seen quickly and the pathway is highly regarded by them, their supporters, and referring GPs.

Using the seven ‘service implementation’ outcomes of feasibility, fidelity, penetration, acceptability, sustainability, uptake and costs described by Proctor et al. [17], the RDC implementation studied here has demonstrated to be a feasible service having been successfully rolled-out across four localities in seven months to include all GP practices. Available clinic slots are all routinely occupied; and we found high awareness and support among GPs about the service. Its take-up is evenly spread across the four UHB localities. Service referral protocols are followed and, where necessary, have been adapted following constructive engagement with primary care. Patients, too, have been supportive in their experience of the clinic as the focus groups showed. Regarding costs, although we were only able to undertake a limited analysis of RDC-specific costs, these were acknowledged to be commensurate with existing local clinic costs and were comparable with findings from the Swansea pilot study [7]. Unfortunately, there were no cost data available to compare with similar RDC-type clinics in England.

### Comparison with existing literature/ studies

A number of similar initiatives have been launched in the UK, Table 6. In London, Guy’s and St Thomas’s NHS Trust (GSTT) was an early adopter of the RDC in the UK. Together with Oxford, this is a Cwm Taf comparator site (discussed below). In England, Cancer Research UK and Macmillan established the Accelerate, Co-ordinate, Evaluate (ACE) Programme of 50 sites trialling an RDC approach focused on cancer detection and diagnosis [18]. A second phase – ACE 2 (or ACE-MDC) – ran in five sites (including Oxford) which included non-cancer diagnoses [19].

**Table 6:** Cwm Taf in comparison with other sites that have implemented a version of the Rapid Diagnostic Clinic

	Cwm Taf	ACE-MDC	Oxford SCAN	GSTT RADC
Cancer diagnosis rate	7.3–12.3%	8% overall (4–11% within sites)	10%	7%
Time to RDC appointment (days)	Mean 13.9 Median 12	Median 8	–	Mean 10.2
Time to start of cancer treatment (days)	Mean 40 Median 34	Median 19	27^a^	Mean 43

*ACE-MDC* Accelerate, Co-ordinate, Evaluate Multidisciplinary Diagnostic Centres, *Oxford SCAN* Oxford Suspected CANcer pathway, *GSTT RADC* Guy’s and St Thomas’s NHS Foundation Trust Rapid Access Diagnostic Clinic

^a^ Unclear whether this figure is for a median or mean number

Initial findings from ACE-MDC [20] indicate similar achievements to Cwm Taf, and similar difficulties in obtaining cancer staging data (noting many cancers only first presenting at an advanced stage, and it is too early to assess if there has been any significant increase in earlier stage diagnoses). In Oxford, for example, only 51% of diagnoses had cancer staging data available, and 61% of patients first presented at a late stage.

Parallels with Cwm Taf are also seen with the Rapid Access Diagnostic Clinic (RADC) clinic at GSTT. This clinic has a larger throughput (30–40 per week), and also accepts direct A&E and GI specialty referrals as well as from GPs. They report (unpublished internal report) 51% of patients had attended their GP more than three times before referral; 48% of patients reported experiencing symptoms for 2–6 months and 38% more than 6 months.

Patient satisfaction was high both in Oxford (70%) and GSTT (91%) as was that of GPs (Oxford 90%; GSTT 84%).

An important caveat is that although the metrics presented are similarly described, there was insufficient information to ascertain exactly how data were recorded (e.g. definition of eligible RDC population), or how some measures were calculated locally (e.g. recording time period).

In comparison to site-specific cancer pathways, our estimate of cancer diagnosis rate is in line with estimates in national studies that reported median rates between 8 and 17.0% across a number of urgent suspected cancer pathways.

The other Wales RDC pilot project at Abertawe Bro Morgannwg UHB began a few months earlier than Cwm Taf and was evaluated after a similar length of time of operation. A very different evaluation approach was adopted here using discrete-event simulation to model a cohort of 1000 patients from referral to radiological diagnosis based on routine RDC and hospital data [7]. These were compared with a control cohort of patients who had been referred to an Urgent Suspected Cancer pathway but subsequently downgraded. Their conclusion was that the RDC for patients presenting with vague or non-specific symptoms suspicious of cancer in primary care reduces the time to diagnosis and provides excellent value for money if run at ≥80% capacity with at least five patients per clinic (£646 per patient). There are currently no comparable cost data available from the ACE-MDC sites in England.

### Limitations

There are several limitations associated with this study. As our data collection and analysis was situated in a single organisation, we do not know whether our findings and conclusions can be generalised in different settings. However, given that many of our findings are in line with studies using different methodologies in different settings, we are confident that the lessons learnt here have a wider applicability. The lack of a control or comparator group of patients limits the conclusions that can be drawn from our study which is a similar issue faced by all other studies that have evaluated comparable new clinic designs. The nature of the study design also limits our capacity to analyse the outcomes of those patients who were diagnosed with cancer, as does the incomplete data on cancer staging (and which we thus we did not report on). Although we made an effort to interview GPs from practices with a range of referral activity and locations (and solicited the opinion of all GPs through the survey), participant bias may be present in the sample of GPs who responded and in the patients who participated in the two focus groups.

### Implications for further research

In line with previous studies, it was a challenge to identify and use a control group for comparison purposes. Future research and evaluation methods can contribute to the evidence base by overcoming this challenge, for example by using a ‘difference-in-differences’ study design [21]. Long-term patient outcomes are unknown and future studies should make an effort to address including long-term cancer survival, and types and outcomes of patients receiving non-cancer diagnoses. Further research is also needed to estimate the costs and benefit implications of extending the RDC model to additional cancer diagnostic pathways.

### Implications for practice

A number of practical recommendations for the RDC service were made during the course of the study arising from observations by the research team as well as from recommendations made by GPs (interviews and survey) and patients (focus groups). These can be grouped under three sub-headings:

Suggestions from patients and supporters

- Further improve liaison and communication with GPs and other hospital departments in terms of passing information back to patients.
- Clarify and formalise the summary of the diagnosis for the patient.
- Expand RDC provision to benefit larger numbers of patients.

Suggestions from GPs

- Improve awareness of the RDC to GPs through a variety of means including:
  - orientation packs for locums and GP trainees, and presentations in the local half-day release training scheme (note, trainees do not practice in the RDC)
  - provision of RDC patient case study vignettes as examples
  - the introduction of a liaison session about the RDC every 6 months for new cohorts of trainee GPs
- Introduce frequent feedback to primary care on RDC performance such as waiting times and outcomes.
- Introduce a simple electronic referral form, accessible from the GP system, with reminders about tests and that could be auto-populated with patient details (as currently happens with other hospital referrals).
- Extend the clinic for the rapid diagnosis of different diseases (e.g. pulmonary fibrosis or abdominal pain).

Suggestions from the evaluation team

- Improve the routine collection, timeliness and quality of data to enable greater ease of reconciliation between the different datasets and more up to date information on cancer diagnoses and their staging.
- Introduce, as part of routine practice, a generic multi-attribute instrument for measuring health status (such as EQ-5D, in line with NICE guidance).
- Undertake a detailed cost analysis at the local Health Board level between selected RDC and existing USC pathways and other, non-cancer pathways.

## Conclusions

Although it is still too early to draw firm conclusions about long-term cancer survival rates (and cancer staging data are not sufficiently timely or robust to enable conclusions as to indicate any increase in early-stage detection rates), there are positive indicators to suggest that this model of service provision – popular with both patients and clinicians – can set new and higher standards for joint primary and secondary cancer care working and provide a patient-centred care service.

## Supplementary Information

**Additional file 1.**

## Data Availability

Routine activity and patient survey data were made available by Cwm Taf Morgannwg UHB. Data from the GP online survey, individual GP interviews and patient focus groups were recorded by the Evaluation Team and made available to Cwm Taf Morgannwg UHB in a report. Results databases will be handed over to Cwm Taf Morgannwg UHB once the results have been accepted and published in peer-reviewed literature.

## Abbreviations

A&E: Accident & Emergency
ACE: Accelerate, Co-ordinate, Evaluate
ACE-MDC: Accelerate, Co-ordinate, Evaluate - Multidisciplinary Diagnostic Centre
CaNISC: Cancer Network Information System Cymru
CHI^2^: Centre for Healthcare Innovation and Improvement
CT: Computed Tomography
GI: Gastrointestinal
GP: General Practitioner
GSTT: Guy’s and St Thomas’s NHS Trust
IQR: Interquartile Range
MDT: Multidisciplinary Team
NICE: National Institute for Health and Care Excellence
PET: Positron Emission Tomography
RDC: Rapid Diagnostic Clinic
SD: Standard Deviation
UK: United Kingdom
UHB: University Health Board
USC: Urgent Suspected Cancer
UTI: Urinary Tract Infection
