# Supplementary material for: Setting up a rapid diagnostic clinic for patients with vague symptoms of cancer: a mixed method process evaluation study
Source: BMC Health Serv Res. 2021 Apr 17;21:357. doi: 10.1186/s12913-021-06360-0 (PMC8052708; doi:10.1186/s12913-021-06360-0)
Supplement: Supplementary file 1 — Additional file 1. [file 12913_2021_6360_MOESM1_ESM.docx]

Setting up a rapid diagnostic clinic for patients with vague symptoms of cancer: a mixed method process evaluation study

Supplementary material

# A. Online questionnaire used for GP engagement

**EVALUATION OF THE RAPID DIAGNOSTIC CLINIC, CWM TAF MORGANNWG UHB**

GP Online Survey

Cwm Taf Morgannwg UHB is one of two Health Boards piloting the Rapid Diagnostic Clinic (RDC) approach to streamline and speed up the process for patients presenting to their GP with ‘vague symptoms’ that may potentially be indicative of cancer (such as unexplained weight loss, appetite loss, abdominal discomfort or pain, fatigue, general malaise, or GP ‘gut feeling’).

The Board is currently undertaking a rapid evaluation of the impact that the RDC has had since its introduction. This is being carried out by independent researchers from the University of Bath and as part of this evaluation, we are interested in your experiences and views of referring patients to this clinic.

The survey is short and should take less than 10 minutes to complete. When you have completed the survey please click 'Submit'.

Your feedback is anonymous and will be used to inform the evaluation of service as well as recommendations for improvement.

Many thanks for your time and input to the RDC evaluation. We will feedback findings from it in the coming months.

If you would like to know more about this project, or discuss the operation of the RDC in more depth with the team, please don’t hesitate to contact us:

Professor Christos Vasilakis ([c.vasilakis@bath.ac.uk](mailto:c.vasilakis@bath.ac.uk), 01225 383361) Dr Paul Forte

University of Bath April 2019

*Required

# Questions

## 1. In which locality of Cwm Taf is your practice located? *

*Mark only one oval.*

Cynon Merthyr Rhondda Taff Ely

## 2. Approximately how many patients have you personally referred to the Rapid Diagnostic Clinic (RDC) in Cwm Taf Morgannwg UHB? *

*Tick all that apply.*

None

Fewer than 5 patients 5-10 patients

More than 10 patients

## 3. How satisfied are you with the information supplied to you explaining the purpose and functioning of the RDC? *

*Mark only one oval.*

Very satisfied Satisfied Neutral Dissatisfied Very dissatisfied Not applicable

Grateful for any reasons behind your choice

## 4. How clear to you are the criteria for referral to the RDC? *

*Mark only one oval.*

Very clear Clear Neutral Unclear Very unclear

Not applicable

Grateful for any reasons behind your choice

# Page 2

## 5. How convenient do you find the process of referring patients to the RDC? *

*Mark only one oval.*

Very convenient Convenient Neutral Inconvenient Very inconvenient Not applicable

Grateful for any reasons behind your choice

## 6. Have you experienced any problems or significant delays in ordering pre-clinic tests (such as bloods, X-rays, etc.) for your referred patients? *

*Mark only one oval.*

No problems or delays Occasionally

Yes, very often Not applicable

Grateful for any reasons behind your choice

## 7. How satisfied have you been with the speed with which your patient(s) have been seen by the RDC team? *

*Mark only one oval.*

Very satisfied Satisfied Neutral

A little dissatisfied Very dissatisfied Not applicable

Grateful for any reasons behind your choice

## 8. How satisfied have you been with the information you received from the RDC after your patient(s) had been given their diagnosis? *

*Mark only one oval.*

Very satisfied Satisfied Neutral

A little dissatisfied Very dissatisfied Not applicable

Grateful for any reasons behind your choice

# 9. What would you say is the level of awareness of the RDC generally within your practice?

## 9a. Among principal GPs? *

*Mark only one oval.*

Very high High

Neither low nor high Low

Very low

Not applicable

## 9b. Among trainees? *

*Mark only one oval.*

Very high High

Neither low nor high

Low Very low

Not applicable

## 9c. Among locums? *

*Mark only one oval.*

Very high High

Neither low nor high

Low Very low

Not applicable

Grateful for any reasons behind your choice

## 10. What was your experience of managing patients with vague cancer symptoms prior to the introduction of the RDC?

This is an optional question but we are very interested in finding out about your experience prior to the RDC being available and how you managed patients presenting with vague/non-specific symptoms.

11. What would you say is your overall satisfaction with the RDC? *

*Mark only one oval.*

Very satisfied Satisfied Neutral

A little dissatisfied Very dissatisfied Not applicable

Grateful for any reasons behind your choice

## 12. Are there any changes you would like to see that you think will improve the awareness or functioning of the RDC?

**End of survey - thank you!**

# B. Interview guide used for GP engagement

Interview structure

- GP name and GP Practice name [will be anonymised]
- General understanding of the RDC objectives and RDC ‘referral criteria’ (vague symptoms and gut feeling)
- Was there a good briefing about RDC service?
- Reasons you have referred patients to RDC [table 1; all that apply]
- Opinion of the referral criteria – clear/ too vague?
- Which types of pts have you referred to RDC:
  - approx. number/ type of patient (age, other clinical factors/ types of vague symptoms encountered)
- What might you have done before RDC was in place?
- How satisfied are you with the service as a process?
  - ease of making referral/ speed of getting appt/
  - undertaking pre-clinic tests/ admin burden/
  - timely outcome
- Have you found that the clinic referral resolves patient issues?
- Your experience of following up post-RDC:
  - Where patient was triaged out of RDC;
  - Where patient attended RDC and was referred back to GP for treatment;
  - Where patient attended RDC and was referred to a cancer or non-cancer pathway
- Have you received any direct feedback from any patients on their RDC experience? If yes, what was the overall sentiment?
- How has your use of the RDC changed over time (if at all)?
- Do you discuss the RDC/ RDC referrals with GP colleagues?
- What gap in services does RDC address for you?
- In your view what constitutes RDC ‘success’?
- How would you rate your overall experience of the RDC (scale of 1 (bad) -4 (good), and why?
- What changes would you like to see to existing system in short/ longer term?
- Any other views/ comments to add?

Table 1 – tick all that apply

| A)  The symptoms did not fit any 2 week wait criteria or you were concerned that the symptoms might be caused by a malignancy |  |
| --- | --- |
| B) The symptoms were too non-specific to formulate a working diagnosis |  |
| C) You suspected a specific diagnosis but you did not have access to diagnostics in primary care |  |
| D) You were concerned that you were missing a syndrome (‘gut feeling’ |  |
| E) The patient had had multiple attendances |  |
| F)  Your previous interventions had made no difference to the condition |  |
| G)  The patient requested a referral |  |
| H)  You wanted reassurance that you hadn't missed a diagnosis |  |
| I)  Your colleagues advised you to refer the patient to RDC |  |
| J)  You thought this clinic might review the whole patients issues rather than an individual issue/symptom |  |
